# Regulation of PACAP gene expression by ovarian sex steroids and kisspeptin: Study using hypothalamic cell lines and an *in vivo* female rat model

**DOI:** 10.1016/j.bbrep.2026.102834

**Published:** 2026-10-17

**Authors:** Tuvshintugs Tumurbaatar, Aki Oride, Zhuoma Cairang, Hiroe Okada, Haruhiko Kanasaki

**Affiliations:** Department of Obstetrics and Gynecology, Shimane University Faculty of Medicine, Izumo, 693-8501, Japan

**Keywords:** PACAP, Kisspeptin, Estradiol, Hypothalamic-pituitary-gonadal axis

## Abstract

**Objective:**

We investigated the effects of sex steroid hormones on hypothalamic PACAP expression. In addition, the mutual interaction of PACAP and kisspeptin was examined using hypothalamic cell models and an *in vivo* rat model.

**Materials and methods:**

The effects of estradiol (E2), progesterone (P4), and dihydrotestosterone (DHT) on PACAP gene expression was examined using the GT1-7, rHypoE-8, and mHypoA-55 cell lines. The effect of PACAP on Kiss-1 gene expression and the effect of kisspeptin on PACAP gene expression were also examined. In addition, PACAP and Kiss-1 gene expression in the hypothalamus was determined in ovariectomized (OVX) and ovary-intact rats with and without sex steroid hormone supplementation.

**Results:**

PACAP gene expression was not modulated by E2 or P4 stimulation, but DHT stimulation slightly increased its expression in GT1-7, rHypoE-8, and mHypoA-55. Stimulation with 100 nM PACAP significantly increased Kiss-1 gene expression in all three hypothalamic cell lines. Similarly, stimulation with 100 nM kisspeptin-10 also increased PACAP gene expression. PACAP gene expression within the posterior part of the hypothalamus was increased after OVX, and the OVX-induced increase in PACAP gene expression was suppressed by supplementation with E2, P4, and DHT, showing a pattern similar to that observed for Kiss-1 gene expression. In ovary-intact female rats, E2 administration, but not P4 or DHT, reduced PACAP gene expression.

**Conclusion:**

PACAP expression within the hypothalamus may not be regulated directly by E2 and P4. Although PACAP and kisspeptin mutually interact and regulate each other, PACAP expression might be under the strong influence of kisspeptin neurons.

## Highlights

• Hypothalamic PACAP expression was not directly regulated by estradiol or progesterone.
• PACAP and kisspeptin mutually interact and regulate each other within the hypothalamus.
• Kisspeptin neurons may play a dominant regulatory role in modulating hypothalamic PACAP expression.

## Graphical abstract

**Figure:**
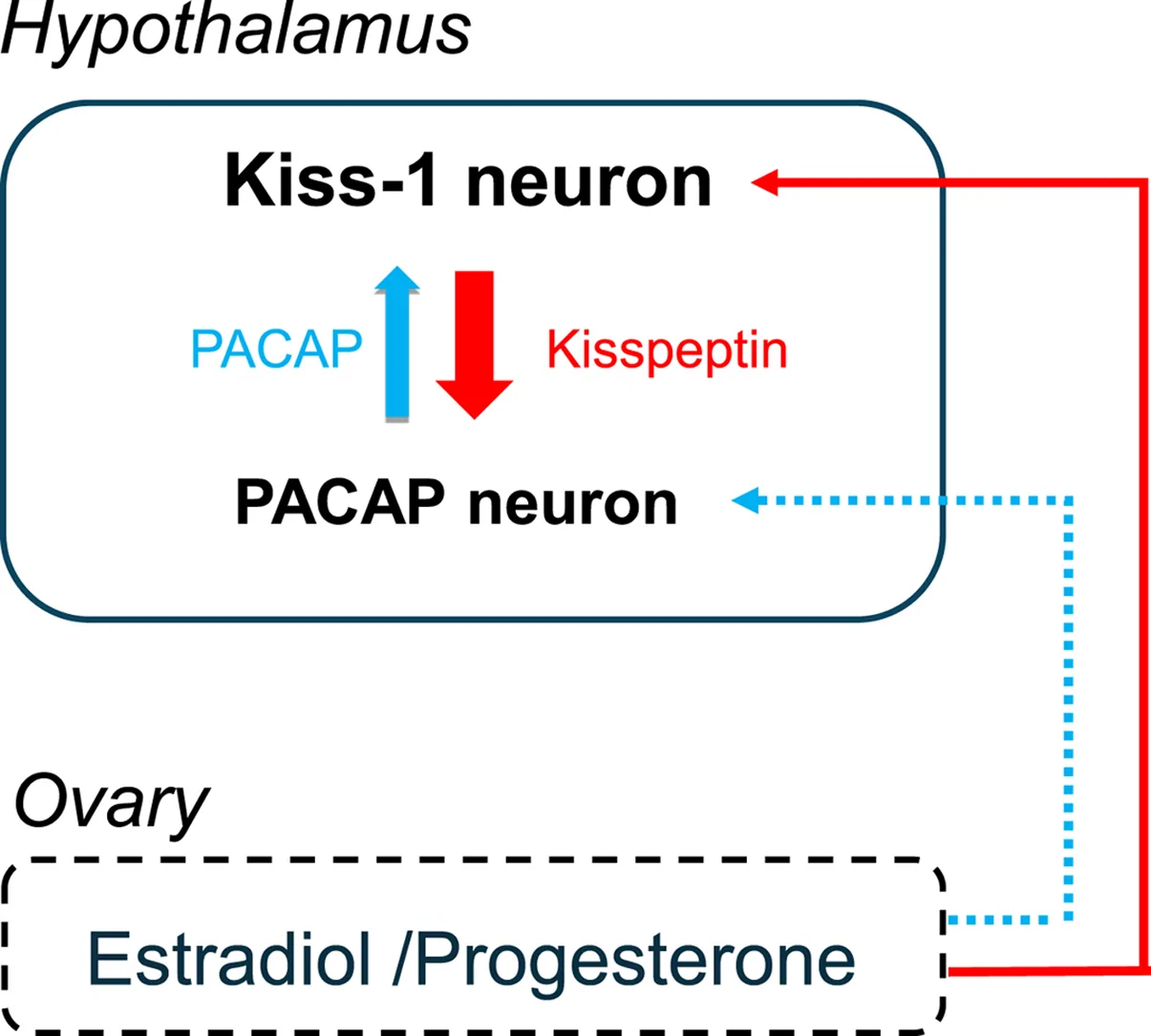

## 1 Introduction

Hypothalamic kisspeptin neurons are recognized as the key regulator of the hypothalamic-pituitary-gonadal (HPG) axis. They act as robust secretagogues of gonadotropin-releasing hormone (GnRH) from GnRH neurons, thereby governing the release of the follicle-stimulating hormone and luteinizing hormone (LH) from the pituitary [1], [2]. Numerous studies have shown that hypothalamus kisspeptin neurons are generally located in the anteroventral periventricular nucleus (AVPV) and arcuate nucleus (ARC) regions [3], [4]. Kisspeptin neurons in the ARC region are called “KNDy neurons” because they co-express kisspeptin, dynorphin A, and neurokinin B, and coordinate their oscillatory activity through autocrine mechanisms involving excitatory feedback mediated by neurokinin B as well as inhibitory feedback mediated by dynorphin A. KNDy neurons in the ARC region are activated by decreasing levels of circulating estradiol (E2) and serve as a center for E2-induced negative feedback regulation, controlling the basal pulsatile secretion of GnRH. In contrast, kisspeptin neurons in the AVPV region are more abundant in females, and are critically involved in mediating an E2-induced positive feedback mechanism. That is, elevated circulating E2 levels during the preovulatory period strongly activate kisspeptin neurons in the AVPV, driving the surge of GnRH that ultimately triggers ovulation [2], [5], [6], [7].

Pituitary adenylate cyclase-activating polypeptide (PACAP) was originally isolated from the ovine hypothalamus and has been reported to stimulate adenylate cyclase activity in the hypothalamus [8]. PACAP is expressed broadly throughout the hypothalamus, including the ARC and AVPV [9], [10], [11]. Moreover, PACAP is detected in the peripheral nervous system and peripheral organs [9], [12], [13], [14]. The potential effects of PACAP and PACAP receptors on reproductive function has been studied extensively, such as PAC1 (PACAP type I receptor) null female mice exhibit significantly reduced fertility compared with their wild-type counterparts [15]. Moreover, subcutaneous PACAP treatment has been shown to delay onset of puberty in rats by affecting the GnRH neuronal system [16], suggesting a critical role in reproductive development. Additionally, the intracerebral treatment of PACAP suppresses LH secretion and ovulation in adult female rats [17], indicating its significant role in modulating reproductive hormone dynamics. Moreover, several studies have demonstrated the potential contribution of PACAP in the regulation of GnRH [16], [18]. It has also been reported that GnRH receptor expression is significantly increased by PACAP treatment in hypothalamic GnRH-producing GT1-7 cells [19]. We have previously reported that stimulation with E2 increases PACAP gene expression in the AVPV-derived kisspeptin-expressing mHypoA-50 cell line, but not in the ARC-derived KNDy neuron mHypoA-55 cell line. Moreover, exogenous PACAP stimulation increases Kiss-1 gene expression in both of these hypothalamic cell lines [20]. These observations suggest that PACAP may have some effect on kisspeptin neurons and influence the HPG axis.

However, it is not clear whether PACAP expression within the hypothalamus is regulated by sex steroids. Moreover, the precise interaction between PACAP and kisspeptin also remains incompletely understood. Therefore, in this study, we investigated the effects of sex steroids on hypothalamus PACAP gene expression. In addition, we examined the mutual interaction of PACAP and kisspeptin, using three different hypothalamic cell lines: GT1-7, rHypoE-8, and mHypoA-55 cells. We also performed *in vivo* experiments involving the hypothalamus of female rats and investigated the changes in PACAP gene expression after ovariectomy (OVX). Finally, we examined the effect of administering sex steroids to ovary-intact rats.

## 2 Materials and Methods

### 2.1 Cell culture and reagents

The rHypoE-8 rat embryonic hypothalamus cell line (COSMO Bio Co., Ltd., Tokyo, Japan), mouse-derived GnRH-producing hypothalamic cell line GT1-7 (donated by Dr. P. Mellon, University of California, San Diego, CA), and mHypoA-55 ARC-derived KNDy neuron cell line (Cedarlane Lab., Burlington, Canada) were treated with water-soluble E2, progesterone (P4), and 5a-dihydrotestosterone (DHT) (Sigma-Aldrich Co., St. Louis, MO); kisspeptin-10 (KP10) (AnaSpec, Fremont, CA). PACAP-38 (pituitary adenylate cyclase-activating polypeptide-38), a 38-amino-acid, C-terminally amidated peptide with the sequence HSDGIFTDSYSRYRKQMAVKKYLAALVLGKRYKQRVKNK-NH_2_, was purchased from Sigma-Aldrich Japan (Tokyo, Japan; Cat. No. A1439; Lot No. 129K4789) and used to investigate the differential regulation of PACAP and Kiss-1 gene expression. These cells were plated individually in 6-well cell culture dishes in high-glucose Dulbecco's modified Eagle's medium containing penicillin-streptomycin (1%) and heat-inactivated fetal bovine serum (10%) (Invitrogen, Carlsbad, CA) at 37°C under a humidified atmosphere of 5% CO_2_ in air until reaching 70%–80% confluency. Then, the cell culture medium was replaced with serum-free high-glucose DMEM and the cells were incubated for 24 h in the presence or absence of stimulation reagents (control).

### 2.2 Western blot analysis

Proteins extracted from the hypothalamic cell lines or fetal rat brain tissues were homogenized in radioimmunoprecipitation assay (RIPA) buffer containing 0.1% sodium dodecyl sulfate, 1% NP-40, and 0.5% sodium deoxycholate in phosphate-buffered saline supplemented with sodium orthovanadate (1 mM), aprotinin (30 mg/mL), and phenylmethyl sulfonyl fluoride (0.1 mg/mL). After homogenization for 10 s, the cell lysates were centrifuged for 10 min at 4°C at 14,000 × *g*. The protein concentration in the cell lysates was measured using the Bradford protein assay. Denatured protein samples in Laemmli buffer were boiled at 95°C for 5 min and 15 μg of protein per well was separated on the 12% sodium dodecyl sulfate–polyacrylamide gel by electrophoresis at 200 V for 35 min, and transferred to polyvinylidene difluoride membranes (Hybond-P PVDF; Amersham Biosciences, Little Chalfont, UK), using semi-dry transfer at 15 V for 30 min. After protein transfer, the membrane was blocked in Blotto (5% milk in Tris-buffered saline) at room temperature for 1 h. Then, the membrane was incubated with an anti-mouse monoclonal PACAP antibody with a dilution of 1:100 (Santa Cruz Biotechnology, Santa Cruz, CA) [21] at 4°C overnight, after which the membrane was washed three times with Tris-buffered saline supplemented with 1% Tween for 10 min per wash. Following primary antibody incubation, membranes were incubated for 1 h at room temperature with the anti-mouse secondary antibody diluted 1:10,000 in Blotto (1% milk in Tris-buffered saline). After enhanced chemiluminescence detection (Amersham Biosciences), the membrane was exposed to X-ray film (Fujifilm, Tokyo, Japan). To correct for protein loading, rabbit monoclonal β-actin antibody at dilution of 1 μg/mL (ab8226; Abcam, Tokyo, Japan) [22] was detected on the same membrane after washing with stripping buffer (sc-281698; Santa Cruz Biotechnology).

Fetal rat brain tissue was used as a positive control. All experimental procedures were approved by the Animal Care and Use Committee of the Experimental Animal Center for Integrated Research at Shimane University (approval no. IZ27-82).

### 2.3 RNA preparation, reverse transcription, PCR, and quantitative real-time (RT)-PCR

Total RNA was extracted from rHypoE-8, GT1-7, and mHypoA-55 cells at 70%–80% confluency and 10–20 mg tissue from the posterior part of the rat hypothalamus, using TRIzol LS (Thermo Fisher Scientific, Waltham, MA). Total mRNA purity was assessed by measuring the A260/A280 absorbance ratio, and the samples with ratios greater than 1.8 were considered acceptable. Measurements were performed using NanoDrop spectrophotometer (Thermo Fisher Scientific). First-strand cDNA was generated from 1.0 μg of total RNA, using an oligo(dT) primer (Promega, Madison, WI) and a First-Strand cDNA Synthesis Kit (Invitrogen) according to the manufacturer's instructions. The final volume of reaction mixture was 10 μL, supplemented with 10 mM dithiothreitol, 200 U RNase inhibitor/human placenta ribonuclease inhibitor, and 1 mM each dNTP (#2310; Takara, Tokyo, Japan). Then, the reaction mixtures were heated at 37°C for 60 min. Quantification of PACAP and Kiss-1 gene expression was determined by quantitative RT-PCR, using a Takara TP900 system (Takara-Bio, Tokyo, Japan) in combination with Universal ProbeLibrary probes (Roche Diagnostics, Mannheim, Germany) and FastStart Master Mix. The PCR primers were designed based on the previously published sequences of PACAP and Kiss-1 [23], and additional primers were designed for GPR54 (Kiss1R) and PAC1R (Adcyap1r1). The primer sequences were as follows: PACAP (amplicon size: 230 bp), forward 5′-GATGTCGCCCACGAAATCCT-3′ and reverse 5′-GTATGCTATTCGGCGTCCTT-3′; and Kiss-1 (amplicon size: 91 bp), forward 5′-ATGATCTCGCTGGCTTCTTGG-3′ and reverse 5′-GGTTCACCACAGGTGCCATTTT-3′; GPR54 (amplicon size: 175 bp), forward 5′-CTGCCACAGACGTCACTTTC-3′ and reverse 5′-ACATACCAGCGGTCCACACT-3′; and PAC1R (amplicon size: 173 bp), forward 5′-TTCACTACTGCGTGGTGTCC-3′ and reverse 5′-TGCACGCAGCTGAAGTAGAT-3′. PCR amplicons were resolved on a 1.5% agarose gel and detected by ethidium bromide staining with cDNA extraction prepared from fetal rat brain tissue as a positive control. To determine PCR efficiency, cDNA was serially diluted 10-fold, following a previously reported method [24]. The simultaneous measurement of target mRNAs and *Gapdh* enabled normalization at the transcript level. Each set of primers included a no-template control. Each PCR reaction had a total volume of 13.5 μL containing 2 μL cDNA (50 ng/μL). The thermal cycling conditions consisted of an initial denaturation at 94°C for 10 min, followed by 40 cycles of denaturation at 94°C for 30 s, annealing at 55°C for 30 s, and extension at 72°C for 30 s. A final extension was performed at 72°C for 30 s, followed by melting curve analysis from 55°C to 95°C. PCR assays were optimized to obtain amplification efficiencies of at least 95%, and only reactions exhibiting efficiencies between 95% and 105% efficiency were included in the subsequent analyses. The relative differences in cDNA concentration between the baseline state and experimental state were calculated using the comparative threshold cycle (Ct) method [23]. For each sample, ΔCt was calculated using the following equation for normalization against the internal control: ΔCt = Ct(gene) − Ct(*Gapdh*). To obtain the difference between the experimental and control conditions, ΔΔCt was calculated as ΔCt(sample) − ΔCt(control). Relative mRNA levels were calculated using the following equation: fold change = 2^ΔΔCt^.

### 2.4 Animal experiments

Six-week-old female Wistar rats (CE-2; CLEA Japan, Tokyo, Japan) were maintained in pairs under controlled environmental conditions, with a 12-h light–dark cycle and the room temperature kept between 20°C and 25°C. Food and water were provided *ad libitum*. To track their estrous cycles, vaginal smears were collected and examined daily at 10:00. Each experimental group consisted of three female rats. Following a 1-week observation period, the animals were allocated to one of nine experimental groups. Four groups underwent bilateral ovariectomy (OVX), and one group underwent a sham (ovary-sparing) surgery and served as the surgical control for the OVX groups. The remaining four ovary-intact groups received sex steroid treatments. All surgical procedures were performed under anesthesia induced by intraperitoneal administration of a mixture of midazolam (2 mg/kg), medetomidine (0.15 mg/kg), and butorphanol (2.5 mg/kg). Following bilateral ovariectomy (OVX), the rats were allowed to recover for 7 days before receiving either a subcutaneous pellet containing 0.25 mg E2 or 50 mg P4 (Innovative Research of America, Sarasota, FL) or daily subcutaneous injections of DHT (25 mg/kg) dissolved in 140 μL sesame oil for 7 consecutive days. For the sham-operated control group, the animals underwent the same surgical procedure used for subcutaneous pellet implantation except that no pellet was implanted. For the DHT treatment group, the corresponding control animals received daily subcutaneous sham-injections of 140 μL sesame oil for 7 consecutive days. Dose selection and treatment duration were decided based on prior reports [25], [26], [27], [28]. For experiments involving ovary-intact rats, sex steroids were administered consecutively for 7 days without performing OVX, while control animals received a daily injection of 140 μL of sesame oil. At the end of sex hormone treatment, the rats were euthanized under isoflurane anesthesia (Fujifilm Wako Pure Chemical Corp., Osaka, Japan), and the whole brain and anterior pituitary gland were collected and the brain bisected along the sagittal plane. One half of each tissue sample was stored for future analyses, and the other half was processed for the present experiments. The hypothalamus and pituitary gland were isolated with reference to a standard rat brain atlas [29] and then separated into anterior and posterior regions by coronal sectioning for further experiments. All experimental procedures were approved by the Ethics Review Committee of the Shimane University Experimental Animal Center for Integrated Research (IZ66-41).

### 2.5 Cell viability test

To evaluate the potential cytotoxic effects of PACAP treatment, cell viability was assessed using a Cell Counting Kit-8 (CCK-8) assay (Dojindo Molecular Technologies, Kumamoto, Japan) according to the manufacturer's instructions. Cells were seeded at a density of 5000 cells per well in 96-well plates and allowed to attach overnight in high-glucose DMEM supplemented with 1% penicillin-streptomycin and 10% heat-inactivated fetal bovine serum (Invitrogen) at 37°C in a humidified atmosphere containing 5% CO_2_. The culture medium was subsequently replaced with serum-free high-glucose DMEM, and the cells were treated with 100 nM PACAP for 24 h under the same culture conditions used for the gene expression experiments. Following treatment, 10 μL of CCK-8 reagent was added to each well and incubated for 3 h at 37°C. Absorbance was measured at 450 nm using a using a DTX880 multimode microplate reader (Beckman Coulter, Brea, CA). Cell viability was evaluated based on the absorbance values relative to those of the untreated control group.

### 2.6 Statistical analysis

All experiments were independently repeated at least three times. For each condition, quantitative RT-PCR analyses were performed using duplicate samples. When confirming mRNA or protein expression, two samples were analyzed, each measured in duplicate. The mean ± standard error of the mean (SEM) was calculated from four resulting measurements. Three mean values were acquired from three independent experiments, and the data were statistically analyzed and the mean SEM calculated. One-way analysis of variance with Bonferroni's *post hoc* test was applied to assess the effects of the two stimulant doses and to analyze *in vivo* data. Differences were considered statistically significant at *P* < 0.05 or *P* < 0.01.

## 3 Results

### 3.1 PACAP expression in three hypothalamic cell lines

In this study, we used three different hypothalamic cell lines. GT1-7 cells are a hypothalamic neuronal cell line derived from GnRH-expressing neurons of transgenic mice, and are used widely as a selective model for GnRH neurons [30]. rHypoE-8 cells are an immortalized hypothalamic neuronal cell line derived from embryonic day-18 rat hypothalamic tissue [31]. mHypoA-55 cells, a model for KNDy neurons, are kisspeptin-expressing hypothalamic neurons originating from the mouse ARC. Previous studies have demonstrated that these cell models express multiple sex steroid receptors and exhibit functional responsiveness to estrogenic, progestogenic, and/or androgenic stimulation [32], [33], [34], [35], [36], [37], [38]. We confirmed that PACAP, PAC1R, and GPR54 mRNA (Fig. 1A) and protein (Fig. 1B) were expressed in all three hypothalamic cell lines.

**Fig. 1:**
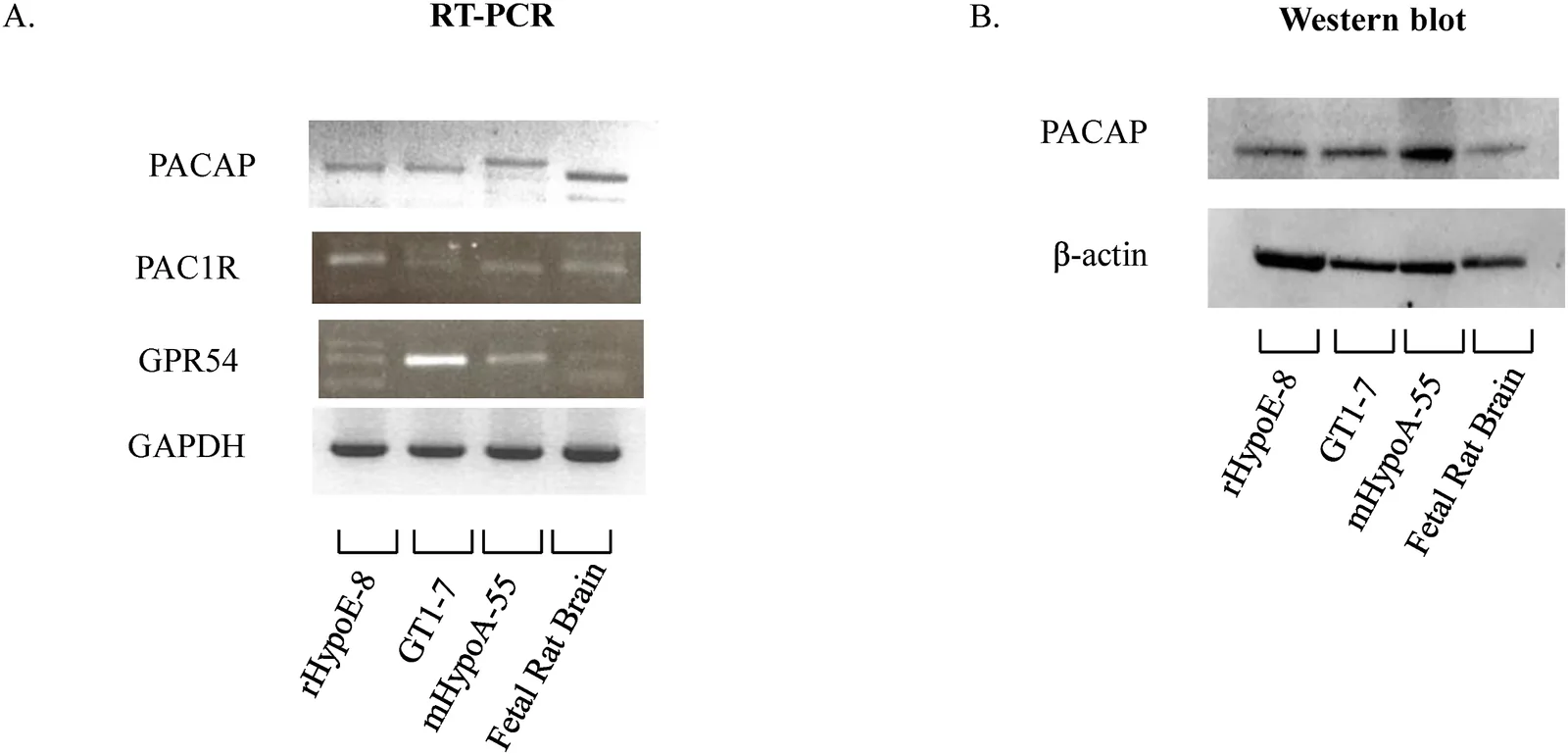
PACAP expression in three different hypothalamic cell lines (A) cDNA was synthesized from 1.0 μg total mRNA extracted from rHypoE-8, GT1-7, and mHypoA-55 cells, with fetal rat brain tissue used as a positive control. RT-PCR was carried out using PACAP-, PAC1R-, and GPR54-specific primers. The amplified products were separated on a 1.5% agarose gel and detected by ethidium bromide staining. (B) Total protein (15 μg) extracted from rHypoE-8, GT1-7, and mHypoA-55 cells as well as fetal rat brain tissue were separated on the 12% sodium dodecyl sulfate–polyacrylamide gel by electrophoresis and followed by 24 h incubation with an anti-mouse monoclonal PACAP antibody. To correct for protein loading, rabbit monoclonal β-actin antibody at dilution of 1 μg/mL was detected on the same membrane after washing with stripping buffer, and fetal rat brain tissues were employed as a positive control. The bands were visualized using a horseradish peroxidase-conjugated anti-mouse secondary antibody.

### 3.2 Effects of sex steroids on PACAP gene expression in hypothalamic cell lines

We investigated the direct effects of sex steroids on PACAP gene expression in the three different hypothalamic cell lines. Stimulation with 10 nM and 100 nM E2 did not induce any significant changes in PACAP gene expression in GT1-7, rHypoE-8, and mHypoA-55 cells (Fig. 2A–C). Similarly, PACAP mRNA expression in these cells was not modulated by 10 nM and 100 nM P4 stimulation (Fig. 2D–F). However, stimulation with 100 nM DHT significantly increased PACAP expression by 2.63 ± 0.55-fold in GT1-7 cells (Figs. 2G) and 1.45 ± 0.27-fold in rHypoE-8 cells (Fig. 2H). Similarly, 10 nM and 100 nM DHT significantly increased PACAP gene expression in mHypoA-55 cells by 1.60 ± 0.10-fold and 1.82 ± 0.15-fold, respectively (Fig. 2I).

**Fig. 2:**
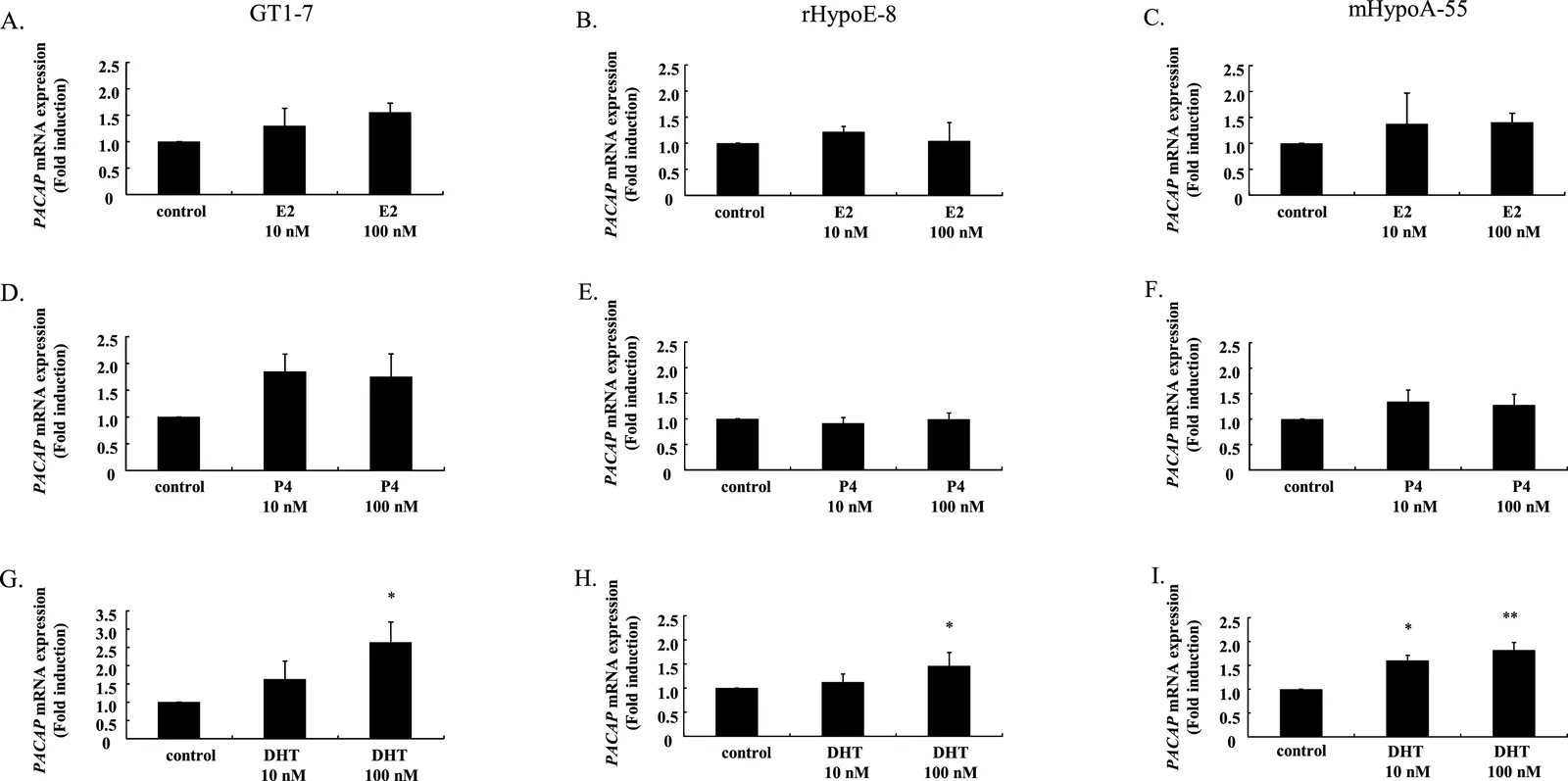
Effect of sex steroids on PACAP gene expression in hypothalamic cell lines GT1-7 (A, D, G), rHypoE-8 (B, E, H), and mHypoA-55 (C, F, I) cells were stimulated with E2, P4, and DHT at concentrations of 10 and 100 nM for 24 h, and first-strand cDNA was synthesized from 1.0 μg of total mRNA. PACAP mRNA expression was measured by quantitative RT-PCR. Results are expressed as fold induction over unstimulated cells and presented as the mean ± SEM values of three independent experiments, each conducted in duplicate. **P < 0.01, *P < 0.05 compared with the control.

### 3.3 Effects of PACAP on Kiss-1 gene expression in hypothalamic cell lines

In addition to PACAP, GT1-7, rHypoE-8, and mHypoA-55 cells express kisspeptin protein and its Kiss-1 gene [20], [39]. We examined the relationship between PACAP and kisspeptin using these three hypothalamic cell lines. In GT1-7 cells, 100 nM PACAP stimulation significantly increased Kiss-1 gene expression by 1.98 ± 0.51-fold (Fig. 3A). Similarly, 10 nM and 100 nM PACAP stimulation significantly increased Kiss-1 gene expression in rHypoE-8 cells by 3.54 ± 0.28-fold and 2.37 ± 0.63-fold, respectively (Fig. 3B). PACAP stimulation also significantly increased Kiss-1 gene expression in mHypoA-55 cells by 1.77 ± 0.38-fold and 2.55 ± 0.14-fold at 10 nM and 100 nM, respectively (Fig. 3C).

**Fig. 3:**
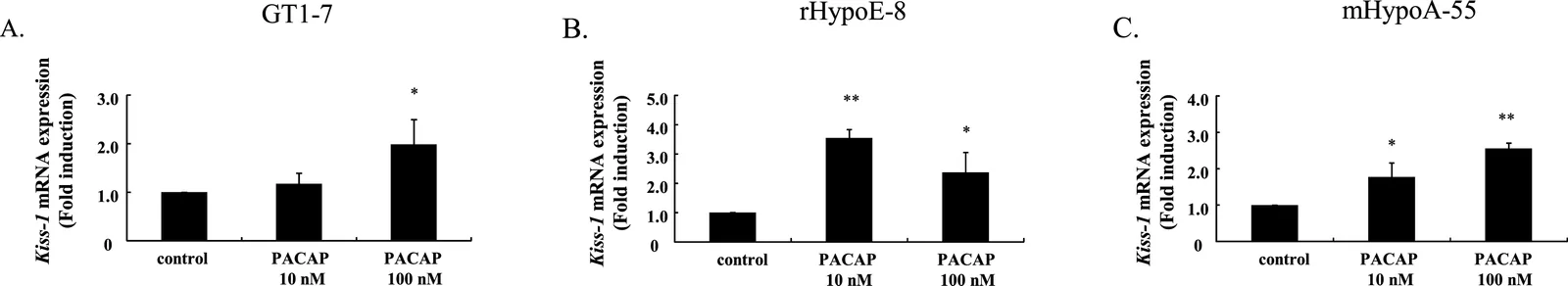
Effect of PACAP on Kiss-1 gene expression in hypothalamic cell lines GT1-7 (A), rHypoE-8 (B), and mHypoA-55 (C) cells were treated with PACAP at concentrations of 10 and 100 nM for 24 h, and first-strand cDNA was synthesized from 1.0 μg of total mRNA. Kiss-1 mRNA expression was measured by quantitative RT-PCR. Data are presented as fold changes relative to untreated cells and are presented as the mean ± SEM of three independent experiments, each conducted in duplicate. *P < 0.05 and **P < 0.01 compared with the control.

### 3.4 Effects of KP10 on PACAP gene expression in hypothalamic cell lines

The effect of KP10 on PACAP gene expression was investigated in the three hypothalamic cell lines. KP10 stimulation increased PACAP gene expression in all three cell lines. In GT1-7 cells, 100 nM KP10 significantly increased PACAP gene expression by 2.13 ± 0.50-fold (Fig. 4A). In rHypoE-8 and mHypoA-55 cells, 100 nM KP10 significantly increased PACAP gene expression by 2.57 ± 0.43-fold (Figs. 4B) and 1.95 ± 0.35-fold, respectively (Fig. 4C).

**Fig. 4:**
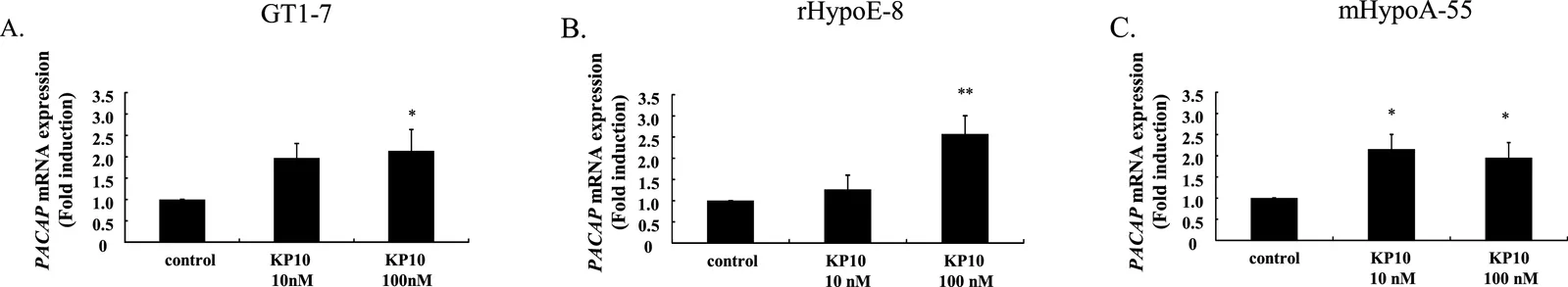
Effect of KP10 on PACAP gene expression in hypothalamic cell lines GT1-7 (A), rHypoE-8 (B), and mHypoA-55 (C) cells were treated with KP10 at concentrations of 10 and 100 nM for 24 h, and first-strand cDNA was synthesized from 1.0 μg of total mRNA. PACAP mRNA expression was measured by quantitative RT-PCR. Data are presented as fold changes relative to untreated cells and are presented as the mean ± SEM from three independent experiments, each conducted in duplicate. *P < 0.05 and **P < 0.01 compared with the control.

### 3.5 Effects of OVX and sex steroid administration on PACAP gene expression in the posterior part of the hypothalamus

OVX has a profound impact on the HPG axis by depleting circulating E2. Therefore, the effects of OVX on PACAP gene expression in the posterior part of the hypothalamus were examined. The posterior hypothalamus was chosen because KNDy neurons, which govern the HPG axis, are located in this area. OVX significantly increased PACAP mRNA expression within the posterior part of the hypothalamus by 1.49 ± 0.32-fold compared with sham-operated control rats. E2 supplementation after OVX was associated with a reduction in PACAP gene expression compared with the OVX group. Similarly, P4 and DHT supplementation after OVX showed a reduction in PACAP gene expression compared with the OVX group (Fig. 5A); however, these reductions did not reach statistical significance. In ovary-intact rats, E2 administration significantly reduced PACAP mRNA expression in the posterior part of the hypothalamus by 0.58 ± 0.08-fold, while P4 and DHT supplementation did not significantly change PACAP gene expression in this area (Fig. 5B).

**Fig. 5:**
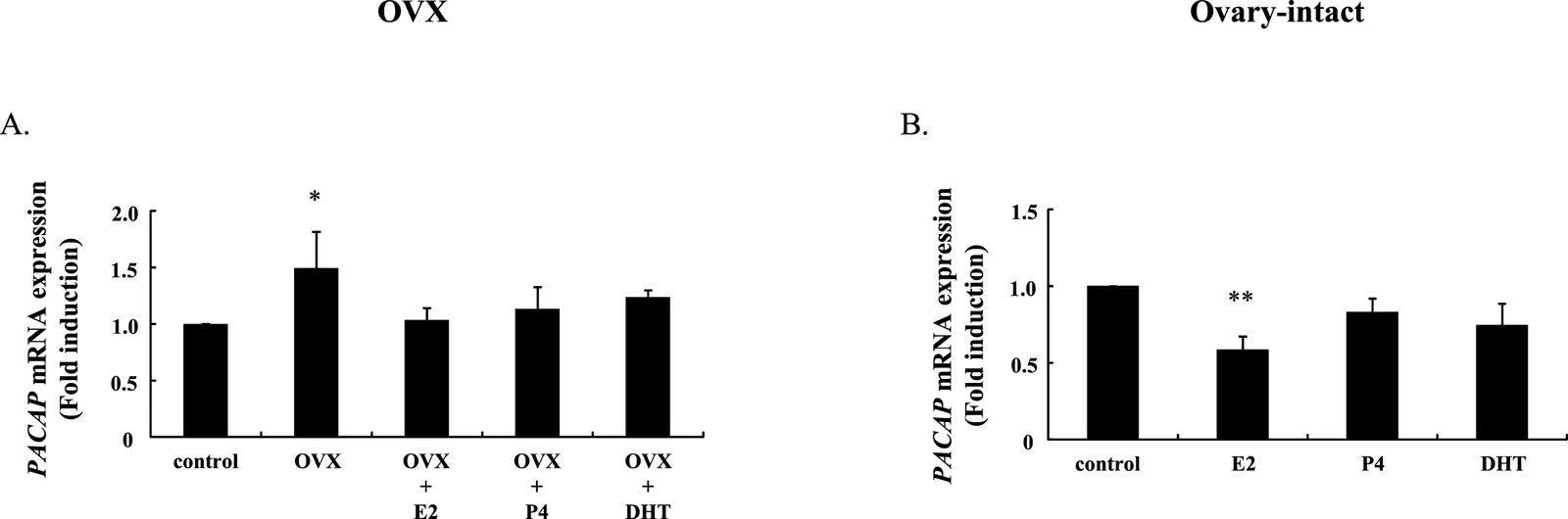
Effects of OVX and sex steroid supplementation on PACAP gene expression in the posterior part of the hypothalamus (A) OVX was performed in female rats at 6 weeks of age. Following OVX, rats were randomly divided into three groups and received either subcutaneous pellet of 0.25 mg E2 (OVX + E2), 50 mg P4 (OVX + P4), or daily subcutaneous injections of 25 mg/kg DHT (OVX + DHT). Sham-operated ovary-intact rats served as a control group. (B) Ovary-intact 6-week-old female rats were randomly divided into three groups and received either subcutaneous pellet of 0.25 mg E2, 50 mg P4, or daily subcutaneous injections of 25 mg/kg DHT. Control rats were administrated 140 μL sesame oil daily. After 7 days of sex hormone treatment, the rats were euthanized under isoflurane anesthesia and the whole brain and anterior pituitary gland were collected for experiments. Then, 1.0 μg of total RNA was isolated from the collected tissues, reverse-transcribed, and used to synthesize first-strand cDNA. PACAP mRNA expression was measured by quantitative RT-PCR. Data are presented as the mean ± SEM obtained from three animals per group. Statistical significance was determined using one-way analysis of variance with Bonferroni's *post hoc* test. *P < 0.05 and **P < 0.01 were considered statistically significant compared with the control. Significant differences were observed between sham-operated (ovary intact) and OVX groups, as well as between ovary-intact and E2-or DHT-treated animals.

### 3.6 Effects of OVX and sex steroid supplementation on Kiss-1 gene expression in the posterior part of the hypothalamus

Finally, the OVX-induced changes in Kiss-1 gene expression in the posterior part of the hypothalamus were examined. As expected, OVX significantly increased Kiss-1 gene expression in this area by 2.63 ± 0.67-fold compared with sham-operated control rats. The OVX-induced increase in Kiss-1 gene expression was almost completely repressed by E2 supplementation after OVX. P4 and DHT supplementation showed a tendency to prevent the increase in Kiss-1 gene expression after OVX compared with the OVX group, although this effect did not reach statistical significance (Fig. 6A). In ovary-intact rats, E2 administration significantly reduced Kiss-1 gene expression by 0.42 ± 0.01-fold, but P4 and DHT had no effect on its expression (Fig. 6B).

**Fig. 6:**
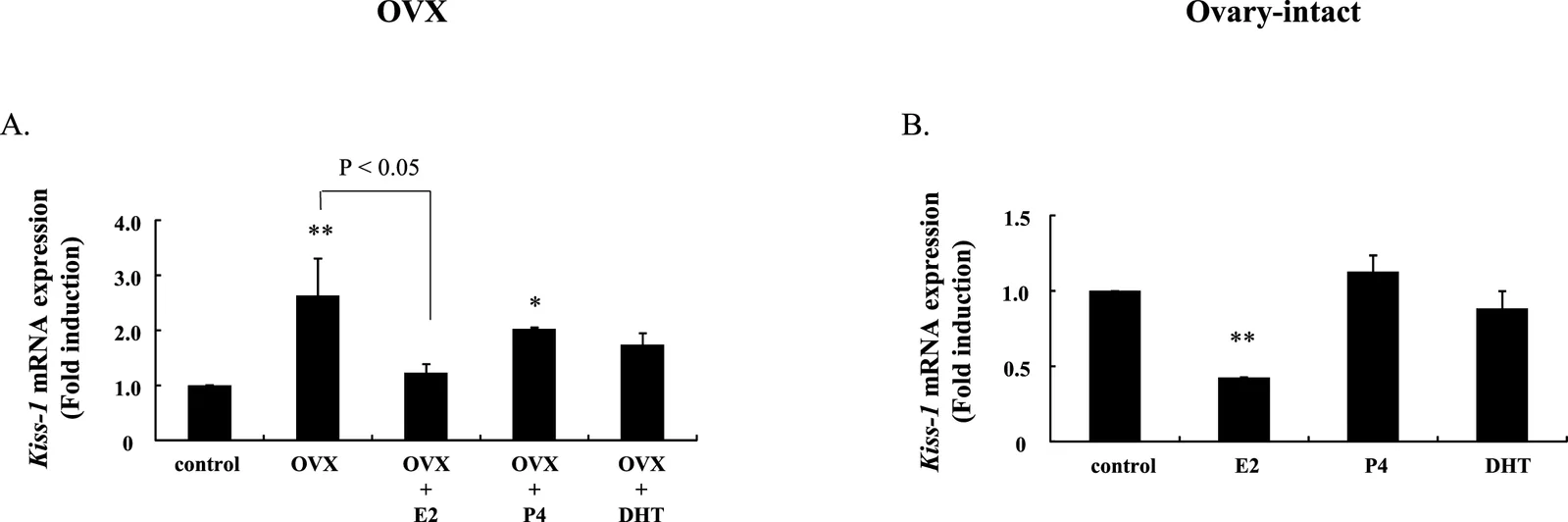
Effects of OVX and sex steroid supplementation on Kiss-1 gene expression in the posterior part of the hypothalamus (A) OVX was performed in female rats at 6 weeks of age. Following OVX, the rats were randomly divided into three groups and received either subcutaneous pellet of 0.25 mg E2 (OVX + E2), 50 mg P4 (OVX + P4), or daily subcutaneous injections of 25 mg/kg DHT (OVX + DHT). Sham-operated ovary-intact rats served as a control group. (B) Ovary-intact 6-week-old female rats were randomly divided into three groups and received either subcutaneous pellet of 0.25 mg E2, 50 mg P4, or daily subcutaneous injections of 25 mg/kg DHT. Control rats were administrated 140 μL sesame oil daily. After 7 days of sex hormone treatment, the rats were euthanized under isoflurane anesthesia and the whole brain and anterior pituitary gland were collected for experiments. Then, 1.0 μg of total RNA was isolated from the collected tissues, reverse-transcribed, and used to synthesize first-strand cDNA. Kiss-1 mRNA expression was measured by quantitative RT-PCR. Data are presented as the mean ± SEM obtained from three animals per group. Statistical significance was determined using one-way analysis of variance with Bonferroni's *post hoc* test. *P < 0.05 and **P < 0.01 were considered statistically significant compared with the control. Significant differences were observed between sham-operated (ovary intact) and OVX groups, as well as between ovary-intact and E2-or DHT-treated animals.

### 3.7 Effect of PACAP on cell viability

To determine whether PACAP treatment affected cell viability, GT1-7, rHypoE-8, and mHypoA-55 were treated with 100 nM PACAP for 24 h, and cell viability was assessed using the CCK-8 assay. PACAP treatment did not significantly affect cell viability compared with the untreated control group (Fig. 7). These results indicate that treatment with 100 nM PACAP for 24 h did not exert detectable cytotoxic effects under the experimental conditions used in this study.

**Fig. 7:**
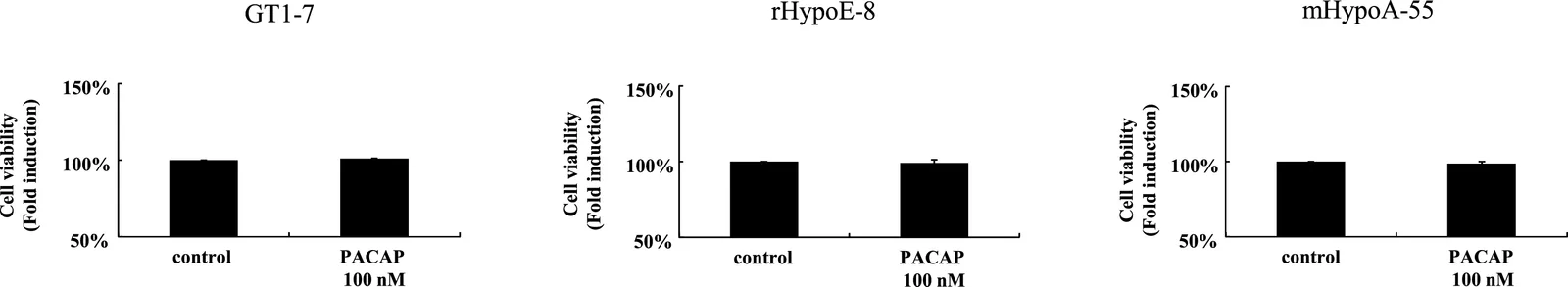
Effect of PACAP treatment on cell viability GT1-7 (A), rHypoE-8 (B), and mHypoA-55 (C) were treated with 100 nM PACAP for 24 h, and cell viability was assessed using the Cell Counting Kit-8 (CCK-8) assay. Absorbance was measured at 450 nm. Data are presented as the mean ± SEM. No significant difference in cell viability was observed between the control and PACAP-treated groups.

## 4 Discussion

In the present study, *in vivo* experiments showed that OVX increased PACAP gene expression in the posterior part of the hypothalamus, which includes the ARC, whereas sex steroid supplementation showed a limited reduction in PACAP expression compared with OVX, although this reduction did not reach statistical significance. Likewise, in ovary-intact female rats, E2 administration significantly suppressed PACAP gene expression. The expression pattern of PACAP mRNA in OVX and E2-supplemented rats completely paralleled the classic Kiss-1 gene expression pattern, in which OVX elevates Kiss-1 gene expression and E2 supplementation suppresses it [2], [5], [32]. In contrast, *in vitro* experiments in three different hypothalamic cell lines—GT1-7, rHypoE-8, and mHypoA-55 cells—showed that PACAP gene expression was not affected by exogenous E2 or P4 stimulation and was only modestly increased by DHT. Importantly, we found an interaction between PACAP and kisspeptin: exogenous PACAP stimulation significantly increased Kiss-1 expression in all three hypothalamic cell lines, while kisspeptin (KP10) increased PACAP gene expression in these cells. Thus, it is conceivable that PACAP and kisspeptin are mutually upregulated by each other. From our present results, we speculate that PACAP may not be a direct target of sex steroids and that kisspeptin signaling may contribute to the regulation of PACAP expression. Moreover, we observed that the increase in PACAP expression following OVX likely reflects the concurrent rise in kisspeptin.

Although it is well known that PACAP influences the activity of GnRH neurons [16], [40], [41], its relationship with kisspeptin neurons is poorly understood. Ross et al. showed that PACAP-expressing neurons in the ventral premammillary nucleus project monosynaptically to kisspeptin neurons in the ARC and AVPV regions of the hypothalamus, and that the loss of PACAP in these neurons delays the onset of puberty in female mice [42]. In the present study, we confirmed that OVX increases Kiss-1 gene expression in the ARC and E2 suppresses it [2], [5], [32], and we found that hypothalamic PACAP expression following OVX and E2 supplementation after OVX shows a similar pattern to that of Kiss-1 gene expression. To our knowledge, this parallel regulation of PACAP and Kiss-1 gene expression by OVX *in vivo* has not been described before. In our experiments using three different hypothalamic cell lines, E2 and P4 had no direct effect on PACAP expression. Therefore, we speculated that sex steroids may control PACAP indirectly via Kiss-1 neurons rather than by acting directly on its gene expression *in vivo*.

Our present results showed that PACAP stimulation increases Kiss-1 gene expression in three hypothalamic cell lines. Similarly, we previously reported that PACAP significantly increases endogenous Kiss-1 expression in the mHypoA-50 AVPV-derived kisspeptin-expressing cell line [20]. These observations suggest that PACAP might also influence GnRH neurons thorough kisspeptin neurons. However, we assumed that PACAP might act downstream of kisspeptin *in vivo*; therefore, the OVX-induced upregulation of PACAP could be a consequence of elevated kisspeptin levels. This hypothesis is based on our *in vitro* findings that KP10 significantly increases PACAP gene expression in the three hypothalamic cell lines used in these experiments. Our findings highlighted a bidirectional regulatory loop between PACAP and Kiss-1; however, an E2 milieu *in vivo* seems to have a significant impact on PACAP neurons via kisspeptin neurons. A recent study reported that the PACAP neuron-specific knockout of estrogen receptor alpha did not alter the timing of puberty [43], suggesting that the effect of E2 on the HPG axis is mediated upstream of PACAP, which supports our hypothesis that PACAP is not affected directly by E2.

The present study suggests that following a decrease in E2 levels during the follicular phase or after OVX, kisspeptin output is increased in the ARC, which then promotes PACAP expression in neighboring or connected neurons. DHT increased PACAP gene expression in the hypothalamic cell lines, but it did not alter PACAP expression *in vivo*. At present, it remains unclear whether DHT affects PACAP expression in the hypothalamus. DHT administration prevented the OVX-induced increase in PACAP expression. Furthermore, DHT supplementation to ovary-intact rats had no effect on PACAP expression within the hypothalamus. Although the action of DHT on PACAP may change in the presence of E2, these *in vivo* observations suggest that DHT may not be significantly involved in regulating PACAP expression in the hypothalamus.

We acknowledge several limitations in this study. The results do not directly prove that kisspeptin increases PACAP gene expression *in vivo*. To demonstrate the direct effect of kisspeptin on hypothalamic PACAP expression, we need to consider additional experiments such as intraventricular kisspeptin administration or the use of a kisspeptin receptor antagonist after OVX. It also remains unclear whether kisspeptin unilaterally regulates PACAP *in vivo*. Indeed, it has been reported that PACAP-knockout mice exhibited a decrease in hypothalamic Kiss-1 gene expression [44]. Furthermore, although E2 did not affect PACAP gene expression in the three hypothalamic cell lines used in the present study, we have reported that E2 increases PACAP gene expression in AVPV-derived mHypoA-50 cells [20]. The response to E2 may vary depending on the type of PACAP-expressing cell. Because our *in vivo* hypothalamic sample contained mixed neuronal populations, changes in PACAP mRNA in these samples will reflect an average change across cell types. Likewise, our hypothalamic cell lines have a limitation in that they are immortalized cells originating from different parts of the hypothalamus in different species and may differ from actual hypothalamic neurons in their responsiveness to steroid hormones. It is necessary to investigate the actual changes within hypothalamic tissue following kisspeptin or PACAP administration using immunostaining or *in situ* hybridization.

## 5 Conclusion

The findings of this study suggest that hypothalamic PACAP might be indirectly regulated by sex steroids via kisspeptin neurons, rather than by direct steroid feedback. The mutually reinforcing loop between PACAP and kisspeptin may influence GnRH neuron activity; however, at least in the context of a dramatic reduction of E2 such as after OVX, the increase in PACAP within the hypothalamus is thought to accompany the increase in hypothalamic kisspeptin. This work focused on PACAP as a potential downstream effector of steroid-sensitive kisspeptin neurons, adding new insights into the neuroendocrine control of female fertility. Further *in vivo* studies directly manipulating kisspeptin signaling are needed to determine whether kisspeptin neurons mediate the regulation of hypothalamic PACAP by ovarian sex steroids.

## Ethics declaration

This study was conducted in accordance with the ARRIVE (Animal Research: Reporting of In Vivo Experiments) guidelines. This study was approved by the Ethics Review Committee of the Shimane University Experimental Animal Center for Integrated Research. (Approval No. IZ66-41; IZ27-82)

## Author's contributions

T.T. and H.K. designed the study; T.T., A.O., Z.C., and H.O. conducted the research and analyzed the data; T.T. and H.K. wrote the paper; H.K. supervised the study.

## Ethical approval

The animal study protocol was approved by the Ethics Committee of the Experimental Animal Center for Integrated Research at Shimane University (IZ66-41 and IZ27-82).

## Consent to publication

All listed authors have reviewed and approved the manuscript for publication.

## Funding

This work was supported by Grants-in-Aid for Scientific Research from the 10.13039/501100001700Ministry of Education, Culture, Sports, Science and Technology of Japan (Grant numbers: 23K08798 and 25K20090).

## Declaration of competing interest

The authors declare that they have no known competing financial interests or personal relationships that could have appeared to influence the work reported in this paper.

## Data Availability

Data will be made available on request.
